# Ultrafast light induced unusually broad transient absorption in the sub-bandgap region of GeSe_2_ thin film

**DOI:** 10.1038/srep03686

**Published:** 2014-01-14

**Authors:** A. R. Barik, Mukund Bapna, D. A. Drabold, K. V. Adarsh

**Affiliations:** 1 Department of Physics, Indian Institute of Science Education and Research, Bhopal 462023, India; 2 Department of Physics and Astronomy, Ohio University, Athens, Ohio 45701-2979, USA

## Abstract

In this paper, we show for the first time that ultrafast light illumination can induce an unusually broad transient optical absorption (TA), spanning of ≈ 200 nm in the sub-bandgap region of chalcogenide GeSe_2_ thin films, which we interpret as being a manifestation of creation and annihilation of light induced defects. Further, TA in ultrashort time scales show a maximum at longer wavelength, however blue shifts as time evolves, which provides the first direct evidence of the multiple decay mechanisms of these defects. Detailed global analysis of the kinetic data clearly demonstrates that two and three decay constants are required to quantitatively model the experimental data at ps and ns respectively.

Chalcogenide glasses (ChG) composed of group six elements except oxygen as major constituent, exhibit unique photoinduced effects when illuminated with bandgap or sub-bandgap light^1^,^2^,^3^,^4^,^5^,^6^. It is believed that photogenerated carriers strongly couple to lattice through phonons and generate defect pairs, which accounts for many of the unusual photoinduced optical and electronic properties of ChG^7^. In ChG, these photoinduced defect pairs are 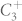 and 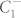 (known as VAPs) where C, super and sub scripts denote the chalcogen atom, charge state and coordination number respectively and forms states near to the conduction and valence band respectively. When 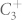 and 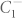 pairs are formed adjacent to each other, they are called as intimate valence alteration pairs (IVAPs). Interestingly light illumination create IVAPs which can annihilate quickly and restore the original configuration or at some times, one of the defect pair can diffuse away through bond switching owing to the structural flexibility associated with the glassy network and form VAP^8^. As a result, light can induce a wide range of physical phenomena through structural rearrangement^9^,^10^. A principal example of such phenomenon is photodarkening (PD), and can be effectively used to prepare intelligent materials possessing certain self control mechanisms which find many potential applications in optics and optoelectronics^11^,^12^,^13^.

Recent first principle molecular dynamic simulations in ChG have shown that light induced structural changes through defects occur at short (<500 fs) to long timescales (>500 fs)^10^. In short time (ST), excited electron transfers energy to lattice and forms unstable IVAPs (defect creation) and in long time (LT), some of these defects are converted into VAPs through bond switching (defect migration and formation of VAPs)^9^,^14^. Quite naturally, for short time illumination, annihilation of defects are more probable, however on the other hand, prolonged illumination favors defect migration through bond switching which eventually will transform a local event into a spatially non-local one at the macroscopic scale^9^,^10^. However, when the light is switched off, some bond switches are frozen in, and give rise to metastable effects. At this point, we define the annihilating defects that decay once the illuminating light was switched off as light induced transient defects (LITD) and the defects which were frozen and needs external stimulus like heat to anneal as light induced metastable defects (LIMD). Notably, in all previous experiments, light induced structural changes were measured after prolonged excitation and hence information is available only about LIMD^2^. Strikingly, there is no experimental evidence available on LITD, more specifically, creation of these defects in short time scale, dynamics involved in stabilization through bond switching in the long time scales and finally the annihilation of defects, though theoretical and computational models predicts their existence and dynamics. Further, in experiments it is difficult to elucidate information on LITD because of their short life time and fast dynamical processes. In that respect, we propose a novel method to study the energy spectrum and dynamics of LITD using an ultrafast pump and white light probe experiment in femto-pico (fs-ps) and nano-micro(ns-μs) second time interval, which falls in the characteristic life time of these defects. The fundamental idea behind our approach is that LITD of different configuration form many localized electronic states in the forbidden gap with a narrow spectrum of energy and optical transitions are allowed between a LITD to either conduction (CB)/valence (VB) band or another LITD. From the observed dynamics, we can discern valuable information on the characteristic relaxation times of LITD and the spectral response provide information on their energy distribution.

In this work, we demonstrate for the first time that ultrafast light illumination can induce an unusually broad TA in a-GeSe_2_ chalcogenide thin film, spanning of ≈ 200 nm in the sub-bandgap region. Our experimental results clearly demonstrate that this broad TA is highly nonlinear in nature, because light illumination creates large number of IVAPs and a fewer number of VAPs at ultra short time scales. Among these IVAPs are short lived and decay within a few 100 s of ps, however on the other hand VAPs have a relatively longer life time and decay within a few 10 s of μs. A detailed global analysis of the kinetic data indicate that two and three decay constants are required to quantitatively model the experimental data at ps and μs time scales respectively and provide new insights on multiple decay mechanisms.

## Results

**Figure 1:**
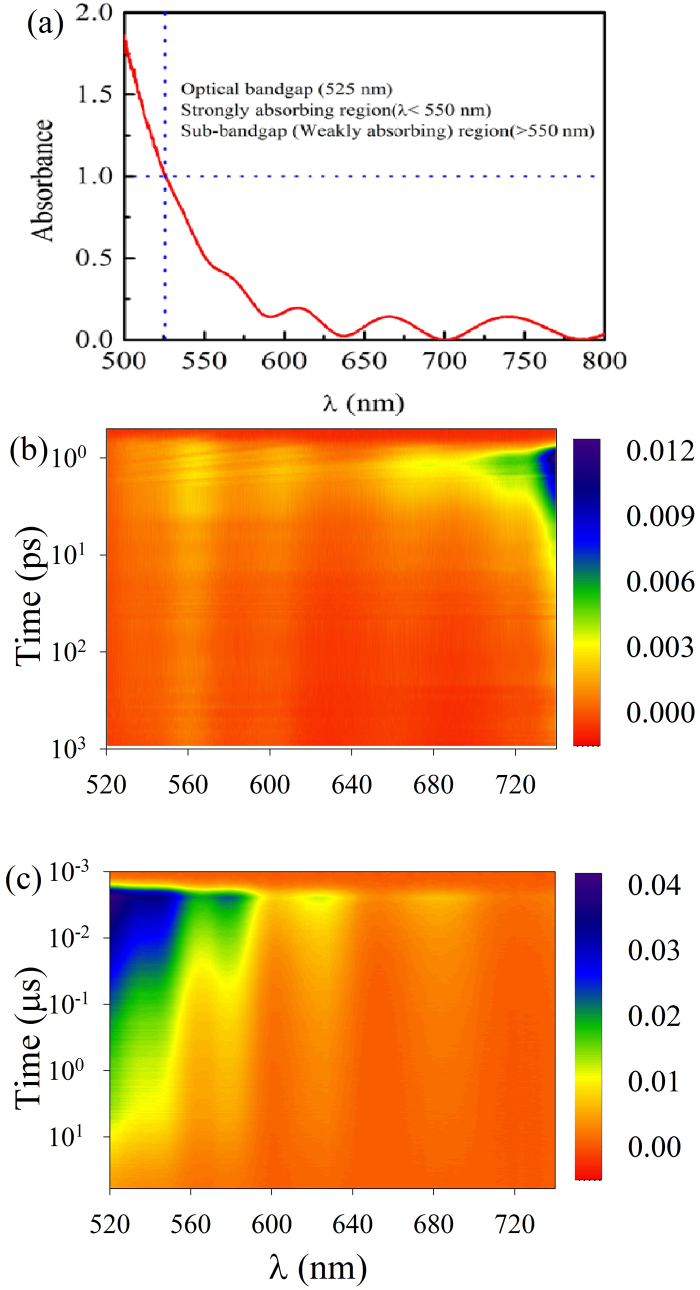
(a) Optical absorption spectrum of GeSe_2_ thin film. Optical bandgap was found to be 525 nm and was determined where Absorbance was one (Absorption coefficient = 10^4^ cm^−1^. Contourplot of time resolved transient absorption (ΔA) spectra of GeSe_2_ thin film in (b) ps and (c) μs time scales. As can be seen from figure that pump beam induces unusually broad TA that extends past 200 nm in the sub-bandgap region.

At first, we have recorded the optical absorption spectrum of as-prepared GeSe_2_ thin film and is shown in figure 1a. To assess the optical bandgap, we have defined it as the energy of photons where the value of absorbance was = 1 (corresponds to an absorption coefficient of 10^4^ cm^−1^). The calculated bandgap value of as-prepared GeSe_2_ thin film is 525 nm (2.36 eV). Below the optical bandgap, there is Urbach region (525–550 nm) where the absorbance show an exponential dependence and sub-bandgap/weakly absorbing region (absorbance < 0.25) where the absorbance is modulated due to interference effects.

**Figure 2:**
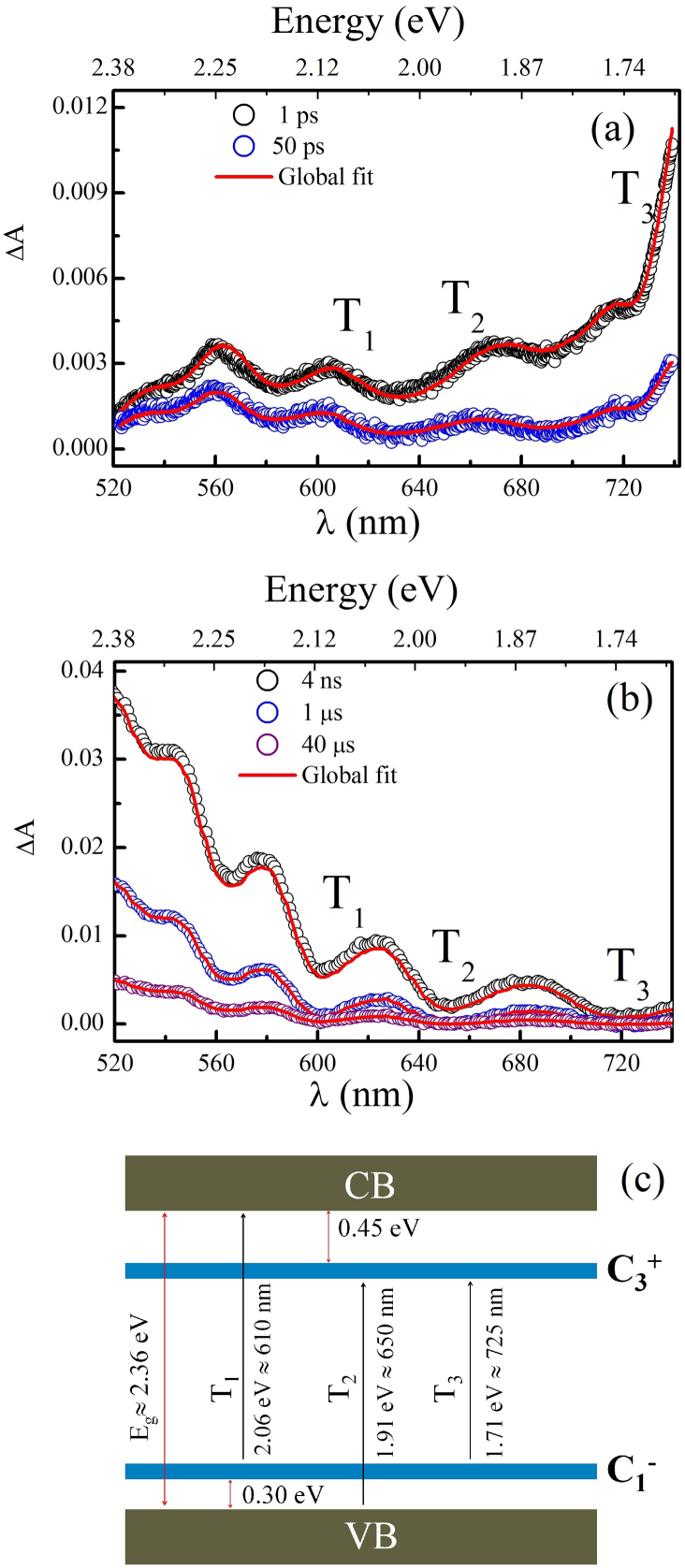
ΔA at various time delays between pump and probe beams for (a) ps (b) μs time scales. Hollow circles and solid red line represent experimental data and global fit (equ. 3) respectively. Interestingly, LITD relaxes within a short time of nearly 100 μs and the absorption maxima peak changes towards shorter wavelength with time evolution. As time evolves, highly unstable IVAPs completely decay and ΔA shift to shorter wavelength in μs time window since major contribution is from VAPs. (c) shows set of possible electronic transitions (T_1_, T_2_, T_3_) in the sub-bandgap region due to LITDs, these transitions lead to several absorption peaks as marked in figure 3(a & b).

To probe LITD, we excited the sample with 100 fs pulses centered at 400 nm and recorded the TA in the wavelength range of 520–740 nm. The overall time resolution of our experiment is better than 130 fs. ΔA of GeSe_2_ thin film immediately following pump beam excitation at two different time scales (ps and μs) are mapped in the contour plot and is shown in figure 1 (b & c). For better understanding, several cross sections of the contour, that is ΔA in spectral range of 520–740 nm at different time delays (ps and μs time scales) are plotted in figure 2 (a&b). As can be seen from figure, following pump beam excitation, there exist a huge residual absorption of probe beam that extends past 200 nm below the bandgap. The most prominent features of TA at two different time scales (ps and μs) are: (1) short lived TA peak at 740 nm and (2) long lived TA band in the wavelength range 520–680 nm.

## Discussion

Explaining and understanding the observed effects is of major importance. In this context, we assumed that ultrafast light illumination induce 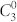 defects. Our idea is based on the predictions by X. Zhang et al, where using *ab initio* molecular dynamic simulations, they have demonstrated that in a-Se, photoexcitation create large number of highly unstable 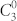 defects^9^,^14^. These highly unstable 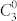 defects try to lower their energy by the charge-transfer reaction^9^,^14^,^15^

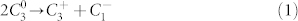
and forms IVAPs. Some of the IVAPs are converted into VAPs, when one of the defect pair diffuse away, however for ultrashort excitation the number of newly created VAPs are very much lower in number than IVAPs. Further, local bonding and mixing between active atomic states of chalcogenide defect and its neighbors will alter the energy levels and as a result they form a broadened rather than a discrete absorption spectrm^15^. For example, M. Cobb et al, using an *ab initio* molecular dynamic simulation have demonstrated that an electronic state crosses the optical gap depending on bonding configurations of a particular atom^16^. In addition to Se atoms, Ge atoms also play a role in bond making and breaking, however less sensitive because of their spherical symmetry^16^. Previous photoluminescence and light induced electron spin resonance (ESR) studies have also indicated that a significant number of VAPs and IVAPs are created by optical excitations^9^,^16^,^17^. Moreover, absence of ESR under dark conditions confirms the idea that 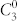 defect is highly unstable and decays into 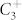 and 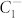 defect pairs. Among, 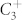 and 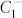 defects, the former one forms localized states near the conduction band (CB) and the latter one forms states near the valence band (VB)^18^,^19^. As a result, under photoexcitation, isolated 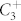 (VAP) act as acceptor and capture a photoexcited electron from VB and similarly an isolated 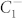 act as donor and donate electron to CB^15^. Strikingly, in a closely spaced 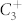 - 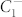 pairs (IVAPs), 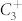 can capture an electron from its nearby 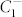, however the optical transitions occurs at lower energies. Figure 2c shows all the possible optical transitions that originate from VAPs, T1(VB to 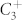 occurs at an energy of 1.91 eV = 650 nm, T2 (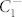 to CB occurs at an energy of 2.06 eV = 610 nm) and IVAPs (T3 from closely spaced 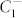 to 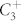 at an energy of 1.71 eV = 725 nm). Among the LITD, IVAPs can self annihilate each other very quickly since 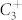 and 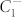 defect pairs are formed too close to each other. However on the other hand, VAPs take a longer time to annihilate since the oppositely charged defect pairs are separated farther apart and as a result they take longer time to come close to each other and neutralize.

After demonstrating the broad TA in the sub-bandgap region which can be divided into fast and slow process on the basis of decay times, we have performed a detailed kinetics analysis of TA measurements to get detailed information on their relaxation dynamics. To model the reaction kinetics, we assume that excitation of ground state leads to population of a particular defect state S_1_. Decay processes of the defect state S_1_ populate many intermediate defect states S_2_ to S_n_, before reaching ground state. Naturally, change in population of a particular LITD can be determined by the rate equation 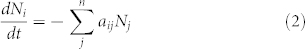
where N_i_ and a_ij_ (i ≠ j) are population density of a state i and rate constants for transition from i to j intermediate states respectively. j can take values from 1 to n depending on the number of intermediate states. General solution of equ.2 is a linear combination of n exponentials and we have fitted the entire spectral and temporal pump probe datasets using a global analysis procedure comprising of multi-exponential function^20^

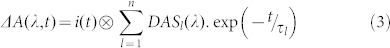
Where *i(t), DAS_l_(λ)*, *τ_l_* and 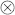 are the instrument response function, termed as decay associated spectra (DAS) and represents the amplitude of the exponential decay function, characteristic time constant for *DAS_l_(λ)* to decay to 1/e of its original value and the convolution operator respectively.

**Figure 3:**
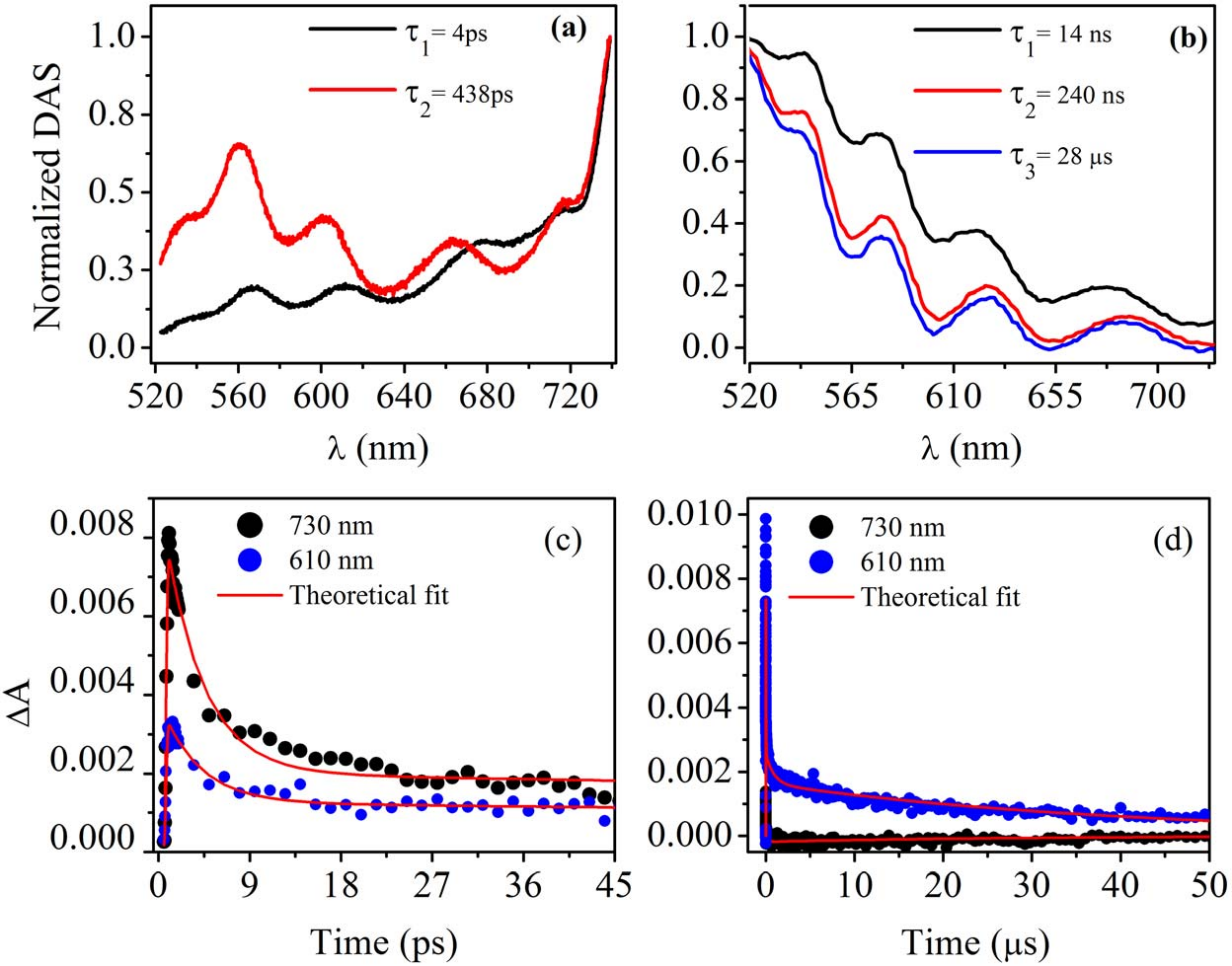
Normalized DAS for different wavelengths (a) ps and (b) μs time scales. From the figures, it is clearly evident that ΔA max blue shift as time evolves. Time dependence of ΔA at 730 nm (due to IVAP's) and 610 nm (due to VAP's) is shown in (c) ps and (d) μs time scales with corresponding theoretical fit. In short time scales (c) IVAP are more in number and decays fast and as a result at longer time scales (d) results in high absorption at 610 nm due to VAPs, since IVAP's decays completely (ΔA = 0 at 730 nm).

First, we will discuss the effects in ps time scale. Best fit to the experimental data at this time scales were obtained with *l* = 2, corresponding to decay constants τ_1_ = 4 ps and τ_2_ = 438 ps. Further, DAS(*λ*) amplitudes resulting from the fit are shown in figure 3a, We term DAS1 and DAS2 as DAS (*λ*) corresponding to decay constants τ_1_ = 4 ps and τ_2_ = 438 ps respectively. Interestingly, DAS1 (fast decay) dominates at longer wavelength; however on the other hand DAS2 (slow decay) is dominant at shorter wavelength. This observation is in accordance with the model proposed by us. From the observed dynamics at ps time scale, it can be concluded that short lived IVAPs are dominant at shorter time scale (ps). Similarly in μs time scales, *l* = 3 model give best fit to the experimental data. In this time scale, we have defined NDAS1, NDAS2 and NDAS3 as DAS(*λ*) corresponding to decay constants τ_1_ = 14 ns, τ_2_ = 240 ns and τ_3_ = 28 μs respectively (see figure 3b). It is important to note that no IVAP transition occurs in this time window and all contributions are from VAPs. Many decay constants for a particular defect level in our experiments clearly indicate that a state can decay through different pathways and provides the first direct evidence of the multiple decay mechanisms of these defects.

To get a complete picture of the decay of VAPs and IVAPs, we have selected two wavelengths at 610 nm (T1 transition) and 730 nm (T3 transition) and fitted the experimental data using eq. 3 (figure 3c&d). The complete decay of T1 and T3 transition occurs in 28 μs and 438 ps respectively. From the observed results, it can be inferred that IVAPs are highly unstable and decay immediately; however VAPs have relatively longer life time and decay within 28 μs. Thus our experimental results demonstrate the first direct experimental observation of creation of defects in ultrashort time scales, fast and slow decays of IVAPs and VAPs respectively and finally their complete annihilation. Importantly, VAPs and IVAPs together span the largest extent in the forbidden gap of GeSe2 thin film and forms broad TA that eventually exceeds dynamic Franz-Keldysh or any other known effect^21^,^22^.

In conclusion, we demonstrate for the first time that an ultrafast light illumination in a-GeSe_2_ thin film induces an unusually broad TA, spanning of ≈ 200 nm in the sub-bandgap region. The observed effects in our experiments exceed dynamic Franz-Keldysh effect in semiconductor crystals or the Stark effect in quantum dots. Moreover, our experiments provide a simple way to modulate optical absorption and have great potential for applications in high-speed optical switching devices. Our idea can further be extended to other widely known chalcogenide glass systems and amorphous semiconductors as well. Unusually broad TA in our experiments is attributed to the LITD, which form many densities of states of varying energy in the forbidden gap. Further, reversibility of TA data clearly demonstrates that there exists a dynamic process which is creating and annihilating the light induced states. A detailed global analysis of the kinetic data provides the first direct evidence of the multiple decay mechanisms of the LITD.

## Methods

### Thin film preparation

Bulk samples of GeSe_2_ were prepared from stoichiometric mixture of high pure (99.999%) Ge and Se powders. The mixture was placed in a quartz ampoule which was evacuated at a vacuum of ≈ 1 × 10^−5^ mbar and sealed. Then the ampoule was kept inside a furnace at temperature of 1000°C and rotated for about 24 h in order to ensure homogeneity of the melt. Bulk glass was then obtained by quenching the ampoule in ice cooled water. Amorphous thin films of GeSe_2_ of ~1.0 μm thickness were deposited on a microscope glass substrate following conventional thermal evaporation method in a vacuum of 5 × 10^−6^ mbar.

### Femtosceond transient optical absorption spectroscopy

For our TA measurement in the ps timescale, 100 fs pulses centered at 800 nm with a repetition rate of 1 kHz, was split into two beams to generate pump and probe. Second harmonics of first beam (400 nm, using BBO crystal) with an average energy of 5 μJ was used as the pump beam. The second beam was delayed with a computer-controlled motion controller and then focused into a CaF_2_ plate to generate a white light continuum (400–1000 nm) and was used as the probe beam. The probe beam was overlapped with the pump beam in the sample, and the change in absorbance of the probe beam ΔA = log[I_ex_(s)/I_0_(s)]-log[I_ex_(r)/I_0_(r)] where s, r, I_ex_ and I_0_ are sample, reference, intensities of sequential probe pulses after delay time τ following excitation by pump beam and in ground state respectively, was measured using a spectrometer (MS 2004, 600 lines/mm diffraction gratings blazed at 600 nm) and Si linear photodiode arrays. Kinetics traces were obtained by varying the path lengths of the probe beam using a micrometer resolution translation stage and was chirp corrected using the procedure reported elsewhere^23^. To avoid any heating effects, the sample was rotated continuously.

### Nanosecond transient optical absorption spectroscopy

Transient absorption measurement in ns time scale was performed with a broadband pump-probe spectrometer EOS (Ultrafast Systems LLC). For this experiment, the pump beam was 40 fs pulses centered at 400 nm with an average energy of 1 μJ. Optical changes of the sample after pump beam excitation was measured by a white light supercontinuum (probe beam) generated from a photonic crystal fiber and required time delays were achieved through a digital delay generator and was detected in a fiber optic coupled multichannel spectrometer. Intensity of probe beam in all our experiments were kept very low such that multi photon/multistep process are avoided.

## Author Contributions

K.V. A conceived the idea. A.R.B. prepared the samples. A.R.B. and M.B. performed all the experiments. A.R.B., M.B., D.A.D. and K.V.A. wrote the paper.
